# From metabolism to neurodegeneration: how microglial functional reprogramming drives neurodegenerative diseases

**DOI:** 10.3389/fnmol.2026.1921079

**Published:** 2026-08-20

**Authors:** Qiaoqiao Cui, Ke Zheng, Qiyun Liu, Lizhen Wang, Yixin Liu, Ruifang Bai, Wei Zhang, Junhong Guo, Xueli Chang, Juan Wang

**Affiliations:** 1 Department of Neurology, The First Hospital of Shanxi Medical University, Taiyuan, Shanxi, China; 2 Clinical College, Shanxi Medical University, Taiyuan, Shanxi, China

**Keywords:** amino acid metabolism, disease-associated microglia, glucose metabolism, immunometabolism, lipid metabolism, microglia, neurodegenerative diseases

## Abstract

Microglia are brain-resident myeloid cells that maintain central nervous system homeostasis and respond dynamically to neuronal injury, protein aggregation, and alterations in the local metabolic environment. Single-cell and single-nucleus studies demonstrate that microglial responses in neurodegenerative diseases are highly heterogeneous and cannot be adequately explained by the classical M1/M2 polarization model. Increasing evidence further indicates that metabolic remodeling is not merely a consequence of activation but a determinant of microglial migration, phagocytosis, inflammatory signaling, redox balance, organelle function, and interactions with surrounding neural cells. In this review, we propose a microglial immunometabolic trajectory framework in which metabolic states are viewed as branching and potentially reversible determinants of cellular function rather than fixed stages of a universal disease pathway. We summarize how glucose metabolism, mitochondrial function, lipid metabolism, amino acid metabolism, lysosomal activity, and redox regulation shape microglial plasticity. We further examine relationships among transcriptionally defined states, including disease-associated microglia, microglia associated with neurodegeneration, lipid-droplet-accumulating microglia, and other disease-enriched populations, while emphasizing that transcriptional similarity does not necessarily imply metabolic function or lineage progression. Comparative evidence from Alzheimer’s disease, Parkinson’s disease, and amyotrophic lateral sclerosis indicates that common metabolic regulators, including HIF-1α, mTOR, PKM2, TREM2, APOE, and NLRP3, exert disease-specific effects with unequal mechanistic support. We further distinguish associative metabolic signatures from intervention-based causal evidence and discuss limitations of animal models, immortalized cell lines, postmortem tissue, and induced pluripotent stem cell-derived microglia. Finally, we highlight the need for cell-specific, state-resolved, and temporally precise metabolic interventions that restore defined microglial functions without compromising physiological immune surveillance.

## 1 Introduction

Population aging is increasing the global burden of neurological disorders, including Alzheimer’s disease (AD), Parkinson’s disease (PD), and amyotrophic lateral sclerosis (ALS) (Arthur et al., 2016; GBD 2021 Nervous System Disorders Collaborators, 2024; Zhu et al., 2024). Although these disorders differ in their predominant proteinopathies, anatomical vulnerability, and clinical manifestations, each involves progressive neuronal dysfunction accompanied by changes in the local immune and metabolic environment. Available treatments provide symptomatic benefit or modify selected pathological processes in subsets of patients, but their capacity to prevent or reverse neurodegeneration remains limited. Identifying mechanisms that determine whether neural-resident immune responses remain adaptive or become dysfunctional is therefore an important priority.

Microglia are long-lived, brain-resident myeloid cells that continuously survey the central nervous system (CNS), respond to neuronal activity and tissue damage, remove cellular debris, and contribute to synaptic and tissue homeostasis (Nimmerjahn et al., 2005). During neurodegeneration, these functions are extensively remodeled. Earlier studies frequently classified activated microglia using a binary inflammatory–reparative or M1–M2 framework (Gao et al., 2025). However, single-cell and single-nucleus analyses have revealed multiple microglial states whose transcriptional profiles vary with age, anatomical region, disease type, genotype, and pathological stage (Hammond et al., 2019; Keren-Shaul et al., 2017; Krasemann et al., 2017). Studies of living human microglia have likewise identified disease-enriched subsets associated with antigen presentation, motility, proliferation, lipid handling, and distinct metabolic programs (Sankowski et al., 2019; Tuddenham et al., 2024). These observations indicate that microglial identities cannot be inferred reliably from a small set of inflammatory markers. Moreover, these transcriptionally defined populations were identified in different diseases, anatomical regions, and experimental systems and therefore cannot be assumed to share the same metabolic program or functional properties.

Immunometabolism provides a mechanistic framework for understanding this heterogeneity. Cellular metabolism is not limited to ATP production but also supplies biosynthetic intermediates, controls redox balance, regulates organelle quality, and generates metabolites capable of modifying signaling and chromatin states (Biswas, 2015; Lauro and Limatola, 2020). In microglia, environmental stress can alter glycolysis, oxidative phosphorylation, the pentose phosphate pathway, lipid uptake and oxidation, cholesterol trafficking, amino acid utilization, and glutathione metabolism (Haney et al., 2024; Ryan et al., 2023; Sabogal-Guáqueta et al., 2023). These changes may support migration, proliferation, phagocytosis, and cytokine production during an acute response (Sadeghdoust et al., 2024). Under persistent pathological stress, however, the same pathways may become insufficient or dysregulated, contributing to mitochondrial injury, lipid accumulation, impaired lysosomal processing, altered glutamate release, oxidative stress, and reduced clearance capacity (Espinoza et al., 2025; Lu et al., 2022).

The relationship between metabolism and microglial function is bidirectional. Protein aggregates, damaged neurons, cytokines, hypoxia, altered nutrient availability, and aging can induce metabolic remodeling as a downstream response. Conversely, experimental manipulation of glycolysis, mitochondrial metabolism, or nutrient-sensing pathways can modify inflammatory signaling and microglial function, indicating that at least some metabolic changes participate causally in state regulation rather than merely marking cellular activation (Miao et al., 2023; Milosevic et al., 2025; Sangineto et al., 2023). Nevertheless, causality is likely to be context dependent. Increased glycolysis may provide rapid energy for early surveillance or aggregate uptake in one setting but sustain inflammatory or epigenetic programs in another. Similarly, activation of mTOR, HIF-1α, or PKM2 may support acute adaptation yet become detrimental when prolonged, whereas TREM2 and APOE can couple lipid sensing and processing to survival, phagocytosis, and disease-associated transcriptional responses (Feiten et al., 2026; Haney et al., 2024; Wang et al., 2020). The functions of these regulators therefore cannot be assigned uniformly as protective or harmful.

Several limitations continue to impede interpretation of this field. Many mechanistic studies use immortalized microglial lines, neonatal rodent microglia, lipopolysaccharide stimulation, or peripheral macrophages, which may not reproduce the metabolic constraints of adult human microglia in neurodegenerative tissue. Transcriptomic enrichment of glycolytic, mitochondrial, or lipid pathways also does not demonstrate metabolic flux, and changes measured in whole tissue cannot necessarily be attributed to microglia. Conversely, postmortem human studies provide disease relevance but are influenced by terminal disease stage, treatment history, tissue quality, and the loss of spatial and temporal information. Human induced pluripotent stem cell-derived microglia and multicellular culture systems improve experimental accessibility but remain developmentally and environmentally distinct from aged brain-resident microglia. These considerations are especially important when evaluating therapeutic targets that are shared with neurons, astrocytes, infiltrating macrophages, or peripheral immune cells (Shokr, 2025).

Current literature has largely examined individual pathways or diseases in isolation, leaving several questions unresolved. It remains unclear which metabolic changes initiate microglial dysfunction, which represent compensatory responses to neuronal injury, and which arise only after prolonged disease. It is also uncertain whether apparently similar metabolic programs have comparable functions across AD, PD, and ALS. For example, HIF-1α-, mTOR-, and PKM2-associated glycolytic responses recur across experimental models, whereas TREM2–APOE-dependent lipid handling is supported most strongly in AD. Iron, glutamate, and ferroptotic stress have particular relevance to PD and ALS, but their cellular sources and disease-stage-specific functions remain incompletely defined.

In this review, we propose a microglial immunometabolic trajectory framework in which “trajectory” denotes branching and potentially reversible changes in cellular function across evolving metabolic demands, pathological stimuli, anatomical environments, and genetic backgrounds, rather than a single irreversible lineage from homeostatic to dysfunctional microglia. We first examine how glucose, lipid, and amino acid pathways influence microglial signaling, organelle function, and cellular behavior. We then compare the shared and disease-specific roles of HIF-1α, mTOR, PKM2, TREM2, APOE, mitochondrial dysfunction, and redox metabolism in AD, PD, and ALS. Finally, we evaluate the evidence distinguishing causal metabolic drivers from reactive changes and discuss the challenges of targeting microglial metabolism with sufficient cell specificity, disease-stage precision, and translational relevance.

## 2 Methods

This narrative review was informed by a structured literature search conducted in PubMed/MEDLINE, Web of Science Core Collection, and Scopus from database inception through July 2026. Google Scholar and the reference lists of relevant original studies and reviews were additionally used for backward and forward citation tracking. Because the review focuses on disease-associated microglial immunometabolism, the search was restricted primarily to Alzheimer’s disease (AD), Parkinson’s disease (PD), and amyotrophic lateral sclerosis (ALS).

Search terms were combined using Boolean operators and included terms related to microglial identity, metabolism, disease, and experimental platforms. The principal search structure was: (“microglia” OR “microglial”) AND (“immunometabolism” OR “metabolic reprogramming” OR “glycolysis” OR “oxidative phosphorylation” OR “mitochondrial metabolism” OR “lipid metabolism” OR “cholesterol” OR “fatty acid metabolism” OR “amino acid metabolism” OR “glutamine” OR “glutamate” OR “tryptophan” OR “arginine” OR “redox” OR “iron” OR “ferroptosis” OR “lysosomal metabolism”) AND (“Alzheimer’s disease” OR “Parkinson’s disease” OR “amyotrophic lateral sclerosis” OR “neurodegenerative disease”). Targeted searches were also performed for HIF-1α, mTOR, PKM2, TREM2, APOE, NLRP3, ACSL1, SLC7A11, single-cell RNA sequencing, single-nucleus RNA sequencing, spatial transcriptomics, human induced pluripotent stem cell-derived microglia, and isotope tracing.

The evidence summarized in this Review encompasses studies investigating microglial metabolic pathways, metabolic regulators, organelle function, and metabolism-associated functional states using human tissues, human-derived cellular models, experimental animal models, primary microglia, and established microglial cell lines. Particular attention was given to studies providing mechanistic insight through direct metabolic analyses, genetic or pharmacological perturbation, cell-type-specific approaches, and functional assessment of microglial activities, including phagocytosis, migration, cytokine production, neuronal injury, and glia–neuron communication. Emerging evidence generated by single-cell and single-nucleus sequencing, spatial transcriptomics, proteomics, metabolomics, and lipidomics was also incorporated when it contributed to understanding microglial heterogeneity or metabolism-associated biological programs. Because transcriptional pathway enrichment does not directly reflect metabolic activity or metabolic flux, transcriptomic findings were interpreted together with complementary functional and metabolic evidence whenever available.

Review articles were primarily consulted to establish the conceptual framework, refine terminology, and identify relevant primary studies, whereas mechanistic conclusions throughout this Review were based predominantly on original experimental research. Greater emphasis was placed on studies in which metabolic alterations could be directly attributed to microglia through cell-specific experimental approaches. Findings derived exclusively from whole-tissue analyses or other neural cell populations were interpreted with caution unless supported by complementary microglia-specific evidence. When multiple publications addressed similar questions, the evidence was synthesized according to methodological rigor and biological relevance.

When findings differed across studies, the available evidence was interpreted in the context of experimental design, including species, disease model, brain region, age, sex, genetic background, pathological stage, stimulation paradigm, microglial preparation, and metabolic methodology. Evidence was synthesized according to the degree of mechanistic support, ranging from descriptive observations to intervention-based and causal studies. To ensure that this Review reflects recent developments in the field, additional literature published between January 2024 and July 2026 was evaluated, with particular attention to studies involving human microglia, patient-derived models, state-resolved multi-omics, metabolic-flux analyses, and investigations linking metabolic reprogramming to microglial function in neurodegenerative diseases. As a narrative review, this article provides a qualitative synthesis of the current literature rather than a quantitative assessment of study quality.

## 3 Microglial state heterogeneity and metabolic plasticity

Microglia are long-lived, brain-resident myeloid cells whose functions are continuously shaped by local neuronal activity, extracellular metabolites, tissue damage, aging, and disease-associated signals (Prinz et al., 2019). In the healthy central nervous system, their highly motile processes support environmental surveillance, removal of cellular debris, synaptic regulation, and rapid responses to tissue perturbation (Nimmerjahn et al., 2005; Paolicelli et al., 2011). These functions require substantial metabolic flexibility because microglia must rapidly adjust energy production, biosynthesis, redox balance, and organelle activity according to changing environmental demands. During neurodegeneration, however, persistent exposure to protein aggregates, damaged neurons, inflammatory mediators, altered lipid availability, and oxidative stress can progressively constrain this adaptive capacity (Sabogal-Guáqueta et al., 2023).

Single-cell and single-nucleus transcriptomic studies have demonstrated that microglia do not respond to disease through a uniform activation program. Instead, multiple states emerge according to disease type, anatomical region, pathological stage, age, and genetic background (Hammond et al., 2019; Sankowski et al., 2019; Tuddenham et al., 2024). Disease-associated microglia (DAM), initially identified in 5xFAD mice, are characterized by the downregulation of homeostatic genes and the induction of pathways related to phagocytosis, lysosomal function, and lipid metabolism (Keren-Shaul et al., 2017; Martins-Ferreira et al., 2025). The TREM2–APOE-associated microglial neurodegenerative phenotype, often termed MGnD, similarly involves loss of homeostatic identity and acquisition of disease-associated transcriptional programs. Lipid-droplet-accumulating microglia (LDAM), identified prominently in the aged brain, exhibit defective lipid handling, increased oxidative stress, and impaired phagocytic function (Marschallinger et al., 2020). Terminally inflammatory microglia have also been described in aged and APOE4-associated Alzheimer’s disease contexts and show reduced responsiveness and impaired Aβ uptake (Millet et al., 2024). These populations share selected inflammatory, lysosomal, and lipid-related genes, but they were identified in different experimental systems and should not be regarded as interchangeable states or as consecutive stages of a universal pathway.

Importantly, transcriptomic identity does not necessarily establish metabolic function. Enrichment of glycolytic, mitochondrial, lipid-processing, or amino acid-related genes may indicate altered metabolic demand, but it does not directly demonstrate pathway flux, substrate utilization, or ATP production. Functional interpretation therefore requires integration of transcriptomics with metabolomics, isotope tracing, extracellular flux analysis, organelle measurements, and cell-specific genetic or pharmacological interventions. Available evidence suggests that metabolic remodeling can both enable and restrict microglial responses. Increased glycolysis may support rapid migration, phagocytosis, or inflammatory signaling, whereas mitochondrial damage, defective cholesterol trafficking, impaired amino acid utilization, and lipid-droplet accumulation may reduce long-term adaptability.

**FIGURE 1:**
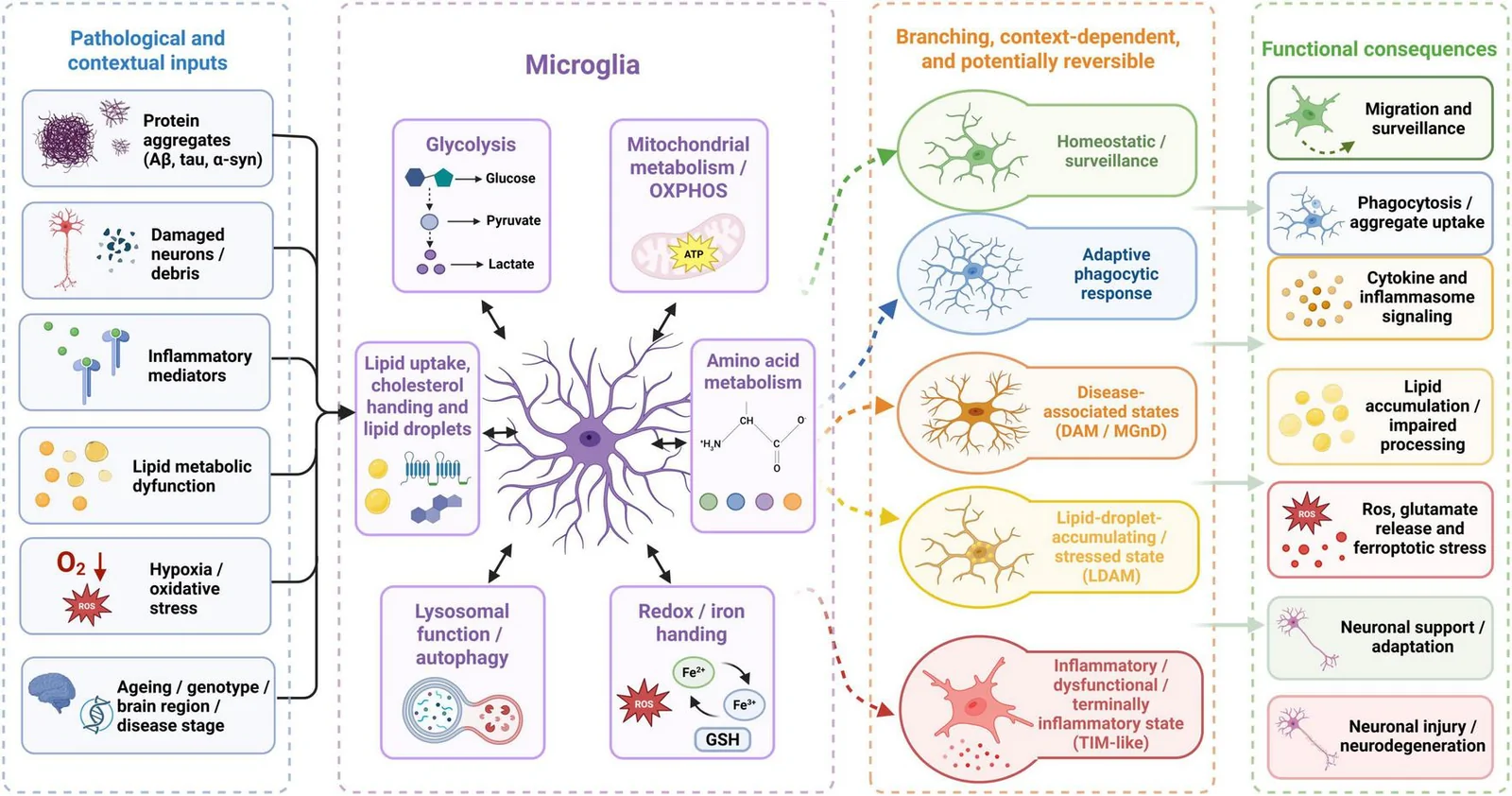
A conceptual framework illustrating microglial immunometabolic trajectories across neurodegenerative diseases. Microglial states are shaped by diverse pathological and contextual inputs, which remodel metabolic programs and drive divergent functional outcomes through dynamic and potentially reversible trajectories.

Accordingly, transcriptionally defined microglial populations should be interpreted together with direct measurements of substrate utilization, organelle function, and cellular behavior. This limitation highlights the need for an integrated framework that connects pathological stimuli, immunometabolic remodeling, and functional state transitions. Rather than representing fixed transcriptional categories, microglial states may be better understood as dynamic and context-dependent immunometabolic trajectories (Figure 1). This framework provides a basis for examining how glucose, lipid, and amino acid metabolism shape specific microglial functions in neurodegenerative diseases.

## 4 Metabolic remodeling shapes microglial functional capacity and plasticity

Microglial functional states emerge from the interaction of extracellular cues, cell-intrinsic signaling networks, and metabolic capacity (Huang et al., 2024). Earlier *in vitro* studies commonly associated LPS/IFN-γ-induced inflammatory responses with increased glycolysis and IL-4-associated responses with greater reliance on oxidative metabolism (Cheng et al., 2021). Although this M1/M2 framework remains useful for describing specific experimental conditions, it does not capture the diversity of microglial states observed *in vivo* and should not be used as a universal classification system. Metabolism is better viewed as a bidirectional component of microglial state regulation: Environmental signals remodel nutrient utilization, whereas the resulting bioenergetic and biosynthetic capacity constrains the functions that microglia can sustain. Consistent with this interpretation, glycolytic inhibition or GLUT1 suppression reduced inflammatory signaling in LPS-stimulated BV-2 cells and primary mouse microglia, while systemic administration of 2-deoxy-D-glucose attenuated microglial activation in LPS- and MPTP-treated mice (Cheng et al., 2021; Wang et al., 2019).

Microglia also exhibit metabolic flexibility rather than dependence on a single energy substrate (Espinoza et al., 2025). Using fluorescence-lifetime imaging and two-photon microscopy in CX3CR1^EGFP/+ mice, acute brain slices, and cultured microglia, Bernier et al. showed that glucose deprivation induces an mTOR-dependent shift toward glutaminolysis that temporarily preserves microglial process motility and damage-sensing responses (Bernier et al., 2020). Conversely, aging and disease-associated stress can impair metabolic flexibility. Microglia in postmortem AD tissue from individuals carrying TREM2 risk variants and in Trem2-deficient 5xFAD mice displayed abnormal autophagic accumulation. Complementary metabolomic and transcriptomic analyses in TREM2-deficient myeloid cells linked this phenotype to impaired mTOR signaling and reduced energetic and biosynthetic capacity, whereas energetic supplementation partially restored plaque-associated microglial responses in Trem2-deficient 5xFAD mice (Ulland et al., 2017). In aged mice, PGE_2_–EP2 signaling reduced glucose flux and mitochondrial respiration in macrophages and microglia, while myeloid EP2 inhibition restored cellular bioenergetics and improved cognitive performance (Minhas et al., 2021).

Accordingly, we conceptualize microglial metabolic remodeling through three interconnected dimensions: Bioenergetic metabolism, which supplies ATP and reducing equivalents; biosynthetic metabolism, which provides substrates for membrane remodeling, inflammatory mediators, and organelle maintenance; and metabolic signaling, through which nutrients and metabolites regulate transcriptional, epigenetic, redox, and stress-response pathways. The balance among these dimensions varies with the initiating stimulus, duration of exposure, brain region, sex, and disease stage. For example, transcriptomic and metabolic analyses of hippocampal microglia from young and aged male and female mice revealed sex-dependent glycolytic rewiring and differential enrichment of disease-associated microglial states (Kang et al., 2024). The relative contribution of bioenergetic, biosynthetic, and signaling metabolism varies among microglial populations and may change over time. The following sections examine how glucose, lipid, and amino acid metabolism influence specific cellular functions and stress responses.

### 4.1 Glucose metabolism

Glucose metabolism supports microglial function by supplying ATP, biosynthetic intermediates, and reducing equivalents. In primary murine brain microglia and commonly used microglial cell lines, several glucose transporters are detectable, but GLUT1 is expressed at the highest level and is further upregulated following LPS/IFN-γ stimulation. Pharmacological inhibition of GLUT1 reduces glucose uptake, glycolytic activity, and inflammatory cytokine production in these experimental systems (Wang et al., 2019). Nevertheless, microglia are not obligatorily dependent on glucose. *In vivo* imaging in mice, acute brain-slice experiments, and cultured microglial systems have shown that glucose deprivation induces an mTOR-dependent shift toward glutaminolysis, which temporarily sustains mitochondrial metabolism, process motility, and damage-sensing functions (Bernier et al., 2020). These observations indicate that substrate flexibility, rather than reliance on a single metabolic pathway, is an important component of microglial homeostasis.

Inflammatory stimulation redistributes glucose flux across glycolysis, mitochondrial metabolism, and biosynthetic pathways. In LPS-stimulated BV2 cells and primary mouse microglia, glycolysis increases rapidly, whereas inhibition of glycolysis with 2-deoxy-D-glucose or 3-bromopyruvate, or knockdown of GLUT1 or HK2, suppresses mTOR–NF-κB signaling and inflammatory mediator production (Cheng et al., 2021). Systemic administration of 2-deoxy-D-glucose also reduced microglial activation and dopaminergic neuronal injury in LPS- and MPTP-treated mice, although these experiments do not establish that the *in vivo* effects were exclusively microglia-mediated (Cheng et al., 2021). Accumulating evidence from multiple *in vitro* studies indicates that activation of the PI3K/Akt/mTOR/HIF-1α pathway is a central regulator of microglial glycolytic reprogramming (Chu et al., 2021; Guan et al., 2023; Li et al., 2024; Zou et al., 2026). Pharmacological or genetic modulation of this pathway alters GLUT1 expression, glycolytic flux, lactate production, mitochondrial function, and inflammatory cytokine secretion in LPS-treated BV2 cells (Milosevic et al., 2025).

Importantly, inflammatory glucose remodeling is not always characterized by a complete replacement of oxidative phosphorylation by glycolysis. In LPS-stimulated HMC3 human microglial cells, both glycolytic activity and mitochondrial respiration increased, but the enhanced respiratory activity was associated with mitochondrial dysfunction and reactive oxygen species generation. Similar bioenergetic alterations were detected in microglia freshly isolated from 3xTg-AD mice. Inhibition of succinate dehydrogenase with dimethyl malonate reduced HIF-1α recruitment, glycolytic activation, and inflammatory responses in HMC3 cells and attenuated brain inflammation in 3xTg-AD mice (Sangineto et al., 2023). These findings indicate that glycolysis and mitochondrial respiration may increase concurrently, while the functional quality and destination of mitochondrial electron flux determine whether oxidative metabolism supports ATP generation or oxidative stress.

Glucose-6-phosphate can also be redirected into the pentose phosphate pathway (PPP), which supplies ribose-5-phosphate for biosynthesis and NADPH for redox reactions. In LPS/IFN-γ-stimulated BV2 cells, increased glycolysis was accompanied by activation of the oxidative PPP while mitochondrial activity remained detectable (Gimeno-Bayón et al., 2014). In primary microglia and multiple experimental PD models, increased expression and activity of glucose-6-phosphate dehydrogenase enhanced NADPH availability for NOX2-dependent reactive oxygen species production and NF-κB activation. Genetic or pharmacological inhibition of glucose-6-phosphate dehydrogenase attenuated microglial activation and dopaminergic neurodegeneration (Tu et al., 2019). Thus, PPP-derived NADPH may support antioxidant systems or, under sustained inflammatory conditions, fuel oxidase-dependent oxidative stress.

Collectively, inflammatory glucose remodeling involves redistribution of carbon among glycolysis, the PPP, and mitochondrial oxidation rather than complete replacement of one pathway by another. The consequences of this redistribution are determined by mitochondrial integrity and by whether glucose-derived metabolites support ATP production, biosynthesis, antioxidant defense, or oxidase-dependent stress. Most mechanistic evidence remains derived from LPS-stimulated cell cultures and rodent models; therefore, whether comparable metabolic fluxes define specific microglial states in the living human brain remains unresolved.

### 4.2 Lipid metabolism

Lipid metabolism supports microglial membrane remodeling, cellular signaling, and the processing of lipid-rich substrates acquired through phagocytosis (Xu et al., 2025). Microglial lipid homeostasis depends on the coordinated balance among lipid uptake, intracellular trafficking, esterification, storage, degradation, and efflux. This balance becomes particularly important during aging and neurodegeneration, when microglia are persistently exposed to myelin debris, apoptotic membranes, and lipid-containing protein aggregates. Depending on the nature and duration of the lipid challenge, lipid storage may initially buffer potentially lipotoxic substrates, whereas impaired processing or persistent accumulation of cholesteryl esters, triacylglycerols, and lipid droplets can restrict phagocytic capacity and promote oxidative and inflammatory dysfunction (Gouna et al., 2021; Haney et al., 2024; Hirsch-Reinshagen et al., 2004; Marschallinger et al., 2020; Nugent et al., 2020; Stephenson et al., 2025).

TREM2-dependent lipid sensing and metabolic adaptation are particularly important when microglia encounter sustained loads of cholesterol-rich material (Gouna et al., 2021; Nugent et al., 2020; Wang et al., 2015). In chronic demyelination paradigms, sorted Trem2-deficient mouse microglia phagocytosed myelin debris but failed to efficiently process myelin-derived cholesterol, resulting in the accumulation of cholesteryl esters. Related abnormalities were reproduced in myelin-treated TREM2-deficient murine macrophages and human induced pluripotent stem cell-derived microglia and were partially corrected by ACAT1 inhibition or LXR activation (Nugent et al., 2020). These findings indicate that TREM2 is involved not only in lipid recognition and uptake but also in the transcriptional and metabolic adaptation required to manage a sustained phagocytic lipid load. In a cuprizone-induced demyelination model, TREM2-dependent cholesterol esterification and lipid-droplet formation in phagocytes were required to limit lipid-induced cellular stress and support subsequent remyelination, demonstrating that lipid-droplet biogenesis can serve an adaptive function under specific conditions (Gouna et al., 2021).

Cholesterol and phospholipid disposal also involves ABCA1-mediated lipid efflux. In cultured astrocytes and microglia derived from Abca1-deficient mice, lipid efflux to APOE was reduced, APOE lipidation was impaired, and intracellular neutral-lipid accumulation increased (Hirsch-Reinshagen et al., 2004). However, because the corresponding *in vivo* experiments were not restricted to microglia, the cell-specific contribution of microglial ABCA1 to neurodegenerative disease remains incompletely defined.

Although transient lipid storage may protect cells from excess free fatty acids and unesterified cholesterol, persistent lipid-droplet accumulation is associated with several dysfunctional microglial states. In aged mouse and human brains, lipid-droplet-accumulating microglia displayed reduced phagocytic activity, increased reactive oxygen species production, and enhanced secretion of inflammatory mediators (Marschallinger et al., 2020). More recent human evidence has linked this phenotype to APOE4-associated lipid dysregulation. Single-nucleus RNA sequencing of postmortem AD brain tissue identified ACSL1-positive microglia that were most abundant in individuals homozygous for APOE4. In human iPSC-derived microglia, fibrillar Aβ induced APOE-dependent ACSL1 expression, triacylglycerol synthesis, and lipid-droplet formation, whereas conditioned medium from lipid-laden microglia increased tau phosphorylation and neuronal toxicity (Haney et al., 2024). Complementary experiments using human iPSC-derived microglia and APOE4-humanized mouse brain slices further showed that direct manipulation of triacylglycerol synthesis altered inflammatory mediator production, phagocytosis, and microglial surveillance-related functions (Stephenson et al., 2025). Together, these findings indicate that lipid droplets are not invariably pathogenic but that their persistence, composition, and turnover can influence microglial functional capacity.

Lipid degradation also depends on peroxisomal pathways. In CRISPR/Cas9-edited BV-2 cells lacking the peroxisomal β-oxidation proteins ABCD1/ABCD2 or ACOX1, accumulation of very-long-chain fatty acids was accompanied by altered expression of lipid-processing, lysosomal, and autophagy-related genes and the emergence of a disease-associated microglial-like transcriptional signature (Raas et al., 2023). Follow-up experiments using the same engineered BV-2 models demonstrated changes in phagocytosis, inflammasome activity, cytokine release, antigen presentation, and responses to LPS stimulation (Tawbeh et al., 2023). These studies provide mechanistic evidence that peroxisomal lipid degradation can influence microglial immune functions, although their relevance to human neurodegenerative disease remains limited by the use of an immortalized murine cell line and models of severe peroxisomal dysfunction.

Collectively, the functional impact of microglial lipid remodeling is determined by the balance between lipid loading and intracellular processing capacity. Lipid uptake or storage becomes detrimental when esterification, lysosomal degradation, peroxisomal oxidation, or cholesterol efflux is insufficient to manage the accumulated substrate (Qiao et al., 2019; Qin et al., 2025; Shokr, 2025; Yildirim-Balatan et al., 2024), New Ref A, New Ref B). TREM2, APOE, lipid-droplet turnover, and organelle integrity therefore influence whether lipid storage serves as a transient buffering response or develops into persistent cellular dysfunction. Evidence from human brain tissue and iPSC-derived microglia is increasing, but most causal interventions remain confined to cell-culture and rodent models.

### 4.3 Amino acid metabolism

Amino acid metabolism regulates microglial function through anaplerosis, redox control, neurotransmitter handling, nitrogen metabolism, and production of signaling metabolites. These pathways do not correspond to fixed inflammatory or reparative phenotypes; their effects depend on the substrate used, the metabolic branch engaged, and the nature and duration of the stimulus.

Glutamine metabolism illustrates this context dependence. Under acute glucose deprivation, *in vivo* imaging in mice, acute brain-slice experiments, and cultured microglial systems showed that mTOR-dependent glutaminolysis sustains mitochondrial metabolism, process motility, and damage sensing (Bernier et al., 2020). In microglia isolated from 8-month-old female APP/PS1 mice and in LPS-primed primary mouse microglia exposed to oligomeric Aβ, glutaminase-1 inhibition reduced glutamine-derived carbon entry into the TCA cycle, activated AMPK, suppressed mTORC1, enhanced mitophagy, and limited NLRP3-dependent caspase-1 and IL-1β activation (Zhang et al., 2024). Conversely, in APP/PS1.Nlrp3-deficient mice and primary murine microglia, NLRP3 loss increased SLC1A3 expression, glutamine and glutamate utilization, mitochondrial respiration, and α-ketoglutarate production. Blocking SLC1A3, glutamate dehydrogenase, or glutamine availability reduced enhanced Aβ uptake, whereas cell-permeable α-ketoglutarate partially restored phagocytosis and influenced chromatin accessibility at phagocytosis-related genes (McManus et al., 2025). Thus, glutamine metabolism may sustain inflammasome activity or support mitochondrial and epigenetic programs that facilitate substrate clearance, depending on pathway utilization (McManus et al., 2025; Zhang et al., 2024).

Cystine and glutamate metabolism links intracellular antioxidant defense to extracellular neuronal stress. System xC^–^ imports cystine for glutathione synthesis while exporting glutamate. In LPS-stimulated primary rat microglia, system xC^–^ inhibition reduced cystine uptake and glutamate release and protected primary cortical neurons in transwell coculture (Gajewski and Barger, 2023). Co-injection of LPS and cystine into mouse spinal cord increased xCT expression in lesion-associated CNS macrophages and produced glutamate receptor-dependent neuronal injury, although resident microglia were not distinguished from recruited macrophages (Kigerl et al., 2012). Metabolomic analyses of purified mouse microglia further identified glutathione and polyamine pathways as microglia-enriched programs. Their abundance declined with aging and in 5xFAD mice, while global Gpx1 deletion altered microglial metabolism and increased Aβ deposition in 5xFAD mice, although the manipulation was not microglia-specific (Yu et al., 2026).

Tryptophan catabolism through the kynurenine pathway generates metabolites with distinct immunological and neuroactive properties. Cultured human microglia convert tryptophan into kynurenine and quinolinic acid, and interferon-γ enhances this pathway (Heyes et al., 1996). In immortalized human microglia, surgical-tissue-derived primary microglia, and human iPSC-derived microglia-like cells, inflammatory stimulation increased IDO1 activity through STAT-related signaling, although responses differed among models (Göttert et al., 2022). KMO inhibition in LPS-stimulated murine microglia and Kmo-deficient primary microglia altered nitrite production and inflammatory mediators (Garrison et al., 2018). In postmortem AD hippocampus, IDO and quinolinic acid immunoreactivity was detected in microglia, astrocytes, and neurons around amyloid plaques, but the contribution of each cell type remains unresolved (Guillemin et al., 2005). The kynurenine pathway should therefore not be considered uniformly neurotoxic.

Arginine metabolism is distributed among nitric oxide, ornithine, polyamine, and argininosuccinate pathways. In an AD mouse model, increased arginine consumption and extracellular arginase activity were associated with reduced brain arginine availability, and combined inhibition of arginase and ornithine decarboxylase attenuated AD-like pathology, although the intervention was systemic (Kan et al., 2015). In Acod1-deficient mice, sorted brain microglia, primary mouse microglia, and BV-2 cells, ACOD1 loss redirected arginine metabolism toward argininosuccinate synthesis, reduced polyamine production, and increased ATP-citrate lyase activity and acetyl-CoA abundance; ATP-citrate lyase inhibition partially reversed these changes (Karadima et al., 2026).

Collectively, amino acid metabolism influences microglial function through TCA-cycle replenishment, antioxidant defense, extracellular glutamate release, epigenetic regulation, and generation of immunoregulatory metabolites. Evidence is strongest for glutamine metabolism in experimental AD, whereas system xC^–^, kynurenine, and arginine pathways remain supported mainly by inflammatory cultures, acute immune-challenge models, or non-microglia-specific interventions. Their roles in reproducible human AD, PD, and ALS microglial states require further validation.

## 5 Microglial metabolic reprogramming in neurodegenerative diseases

### 5.1 Alzheimer’s disease

Alzheimer’s disease (AD) is characterized clinically by progressive cognitive impairment and pathologically by extracellular amyloid-β (Aβ) plaques and intracellular aggregates of hyperphosphorylated tau (Jia et al., 2021; Serrano-Pozo et al., 2011; Terry, 1963). Microglia respond to these lesions through changes in migration, phagocytosis, inflammatory signaling, and metabolic activity. However, available evidence does not support a single metabolic trajectory followed by all AD-associated microglia. Instead, glucose utilization, lipid handling, amino acid metabolism, and mitochondrial function vary with the duration of pathological exposure, APOE genotype, TREM2 signaling, age, and microglial state (Wu et al., 2025; Xu et al., 2025).

Aβ exposure illustrates the temporal and functional complexity of glucose remodeling. In cultured microglia, acute Aβ stimulation induced an mTOR–HIF-1α-dependent increase in glycolysis accompanied by inflammatory activation. Prolonged exposure, in contrast, produced broad defects in both glycolytic and mitochondrial energy metabolism, together with reduced cytokine secretion and Aβ phagocytosis (Baik et al., 2019; Bennett and Liddelow, 2019). RNA sequencing and multiphoton imaging further identified metabolically impaired microglia in 5xFAD mice (Baik et al., 2019). These findings suggest that an initially inducible glycolytic response may become insufficient under chronic Aβ stress rather than remaining constitutively elevated. A separate study using LPS-stimulated HMC3 human microglial cells and microglia isolated from 3xTg-AD mice found concurrent increases in glycolysis and mitochondrial respiration; however, the respiratory response was associated with electron transport chain-derived reactive oxygen species rather than efficient ATP production. Succinate dehydrogenase inhibition reduced HIF-1α recruitment, inflammatory signaling, and metabolic abnormalities in these models (Sangineto et al., 2023). Thus, glycolysis and mitochondrial respiration are not necessarily mutually exclusive, and increased oxygen consumption does not by itself indicate preserved mitochondrial function.

Human tissue provides additional, although primarily associative, evidence. Multiplex immunohistochemistry of hippocampal–entorhinal cortex tissue from patients with AD identified increased numbers of PKM2-positive microglia near Aβ plaques, phosphorylated tau, and cerebral vessels. These cells frequently co-expressed ABCA7 and lipid-droplet markers and showed features interpreted as phagocytic exhaustion. Because this study was based on a relatively small postmortem cohort and did not directly measure metabolic flux, it establishes spatial association rather than demonstrating that PKM2-mediated glycolysis causes microglial dysfunction *in vivo* (Lu et al., 2025).

APOE4 modifies both glucose and lipid responses in microglia. Human iPSC-derived microglia carrying APOE4 showed impaired metabolic activity, migration, and phagocytosis together with increased cytokine secretion, whereas APPswe and PSEN1ΔE9 backgrounds produced different and generally more limited phenotypes in the same experimental platform (Konttinen et al., 2019). In mice expressing human APOE alleles, integrated bulk, single-cell, and spatial transcriptomic analyses, combined with spatial metabolic measurements, identified an APOE4-associated microglial program characterized by increased Hif1α expression, disruption of TCA-cycle-associated pathways, and a pro-glycolytic signature. The magnitude of these changes depended on aging, systemic LPS challenge, and amyloid pathology (Lee et al., 2023). These studies indicate that APOE4 does not impose a single invariant metabolic phenotype but modifies microglial responses to additional environmental and pathological signals.

APOE4 also affects neutral-lipid metabolism. Single-nucleus RNA sequencing of human AD brains identified an ACSL1-positive microglial state that was most abundant in APOE4 homozygotes. In human iPSC-derived microglia, fibrillar Aβ induced APOE-dependent ACSL1 expression, triglyceride synthesis, and lipid-droplet accumulation. Conditioned medium from lipid-laden microglia increased tau phosphorylation and neuronal toxicity, linking microglial lipid remodeling to potentially neurotoxic secreted factors (Haney et al., 2024). Complementary experiments in human iPSC-derived microglia showed that both triglyceride synthesis and triglyceride catabolism were required for activation-associated cytokine production and changes in phagocytosis. In APOE4 microglia, inhibiting triglyceride biosynthesis attenuated disease-associated transcriptional programs and improved surveillance-related behavior in brain slices from APOE4-humanized mice (Stephenson et al., 2025). These intervention studies support a functional role for triglyceride flux, but they also indicate that lipid-droplet formation cannot be classified as uniformly harmful because triglyceride synthesis and turnover may be required for some adaptive responses.

APOE4-associated microglial heterogeneity extends beyond lipid-droplet-containing cells. Microglia-specific deletion of APOE4 restored an MGnD-associated transcriptional response in P301S tau and APP/PS1 mouse models, facilitated plaque-associated microglia–astrocyte communication, and reduced pathology through modulation of an ITGB8–TGF-β checkpoint pathway (Yin et al., 2023). In a longitudinal single-cell atlas of AD-model mice carrying human APOE alleles, aging and APOE4 increased the abundance of terminally inflammatory microglia (TIM). Analogous cells were identified in human AD datasets and APOE4 cortical tissue, and ex vivo assays showed reduced Aβ uptake by TIM-enriched populations (Millet et al., 2024). The metabolic properties of TIM were inferred predominantly from transcriptomic pathway analysis rather than direct isotope tracing or extracellular flux measurements. Accordingly, ACSL1-positive lipid-droplet microglia, MGnD-like cells, and TIM should be treated as distinct but partially overlapping transcriptional populations until their lineage and metabolic relationships are established directly.

TREM2 provides a second major link between lipid sensing and cellular metabolic capacity (Wang et al., 2015). In 5xFAD mice, TREM2 deficiency or haploinsufficiency reduced microglial clustering around Aβ plaques, increased microglial apoptosis, and enhanced Aβ accumulation; the AD-associated R47H variant also impaired recognition of lipid ligands associated with damaged membranes and fibrillar Aβ (Wang et al., 2015). In human AD tissue carrying TREM2 risk variants and in Trem2-deficient 5xFAD mice, microglia accumulated autophagic vesicles. Metabolomic and transcriptomic analyses of TREM2-deficient myeloid cells linked this phenotype to impaired mTOR signaling, reduced ATP availability, and diminished biosynthetic capacity. Dietary cyclocreatine partially restored plaque-associated microglial clustering and reduced plaque-adjacent neuronal dystrophy in Trem2-deficient 5xFAD mice, providing intervention-based evidence that energetic insufficiency contributes to the functional defect (Ulland et al., 2017).

Complementary chronic demyelination experiments showed that TREM2-deficient microglia could internalize myelin but failed to process myelin-derived cholesterol efficiently, resulting in cholesteryl-ester accumulation. Similar abnormalities occurred in myelin-treated TREM2-deficient murine macrophages and human iPSC-derived microglia and were partially rescued by ACAT1 inhibition or LXR activation (Nugent et al., 2020). Although this was not an AD amyloid model, it demonstrates how TREM2 loss can uncouple phagocytic uptake from intracellular lipid processing. A recent TREM2-reporter study in APP-transgenic mice further found that higher TREM2 expression in sorted microglia was associated with oxidative phosphorylation, cellular redox capacity, cholesterol homeostasis, and greater phagocytic activity. Chronic TREM2 agonist treatment produced state-dependent responses, indicating that the effect of TREM2 stimulation depends on the baseline level and state of receptor-expressing microglia (Feiten et al., 2026).

Amino acid metabolism provides an additional link between substrate utilization, mitochondrial function, inflammasome activity, and Aβ clearance. In APP/PS1 mice and LPS-primed primary microglia exposed to oligomeric Aβ, glutaminase-1-dependent glutaminolysis supported NLRP3 activation, whereas glutaminase inhibition activated AMPK, enhanced mitophagy, and reduced inflammasome signaling (Zhang et al., 2024). Conversely, genetic or chronic pharmacological suppression of NLRP3 increased SLC1A3-dependent glutamine and glutamate utilization, α-ketoglutarate production, mitochondrial respiration, and Aβ phagocytosis (McManus et al., 2025). Purified microglial metabolomics additionally identified glutathione and polyamine pathways as microglia-enriched programs that declined during aging and in 5xFAD mice; disruption of glutathione metabolism altered microglial morphology and was associated with increased Aβ deposition, although the genetic intervention was not fully microglia-specific (Yu et al., 2026). These findings show that amino acid-derived carbon can either sustain inflammatory signaling or support mitochondrial, redox, and epigenetic programs, depending on pathway allocation.

Collectively, AD-associated microglial metabolism is shaped primarily by the interaction of Aβ exposure, APOE genotype, TREM2-dependent energetic and lipid handling, aging, and nutrient utilization. These factors differentially influence plaque engagement, intracellular lipid processing, inflammatory signaling, phagocytic capacity, and microglial survival. Human postmortem and iPSC-derived microglial studies have strengthened translational relevance, but direct measurements of metabolic flux in living human microglia remain unavailable. Therapeutic manipulation must therefore account for disease stage and baseline microglial state, because enhancing a pathway that supports acute plaque responses may have different consequences once chronic metabolic stress and functional exhaustion have developed.

### 5.2 Parkinson’s disease

Parkinson’s disease (PD) is characterized by progressive degeneration of nigrostriatal dopaminergic neurons and the accumulation of misfolded and aggregated α-synuclein (α-syn) in Lewy pathology (Goedert et al., 2013; Kalia and Lang, 2015). Microglia participate in the uptake and processing of extracellular α-syn, but chronic exposure to aggregated proteins, inflammatory mediators, mitochondrial stress, and altered iron or lipid availability can impair these responses. Current evidence indicates that microglial metabolic responses to α-syn vary with aggregate structure, exposure duration, inflammatory environment, mitochondrial integrity, and host genotype.

α-Syn exposure can induce both acute metabolic activation and subsequent loss of metabolic responsiveness. In primary microglia exposed acutely to α-syn preformed fibrils (PFFs), increased glycolysis and reduced reliance on oxidative phosphorylation were associated with activation of the AKT–mTOR–HIF-1α pathway. Prolonged PFF exposure instead produced an immune-tolerant state with defects in both glycolysis and mitochondrial respiration, impaired mitophagy, and reduced α-syn uptake. Activation of TRPV1 with capsaicin partially restored metabolism, mitophagy, and phagocytic function *in vitro* (Lu et al., 2022). In mice receiving intrastriatal PFF injections, conditional deletion of Trpv1 in CX3CR1-expressing cells aggravated microglial metabolic defects, α-syn pathology, dopaminergic neuronal loss, and motor impairment (Lu et al., 2022). These findings support a temporal distinction between inducible glycolytic adaptation and chronic metabolic failure, although CX3CR1-driven deletion is not absolutely restricted to long-lived parenchymal microglia.

PKM2 provides a more specific link between α-syn and glycolytic function (Zhang et al., 2025). In primary microglia derived from neonatal rat spinal cord, exposure to wild-type or A53T α-syn increased PKM2 and other glycolytic proteins, lactate production, and cell migration while suppressing mitochondrial biogenesis and oxidative metabolism. PKM2 knockdown reduced glycolysis and migration, whereas the PKM2 activator TEPP-46 enhanced migration (Qiao et al., 2019). Because this study was restricted to a cultured spinal microglial model, it demonstrates PKM2-dependent migration but does not establish enhanced α-syn phagocytosis or a corresponding microglial state in the human substantia nigra.

Human single-nucleus data demonstrate that PD-associated microglia are heterogeneous. Analysis of more than 41,000 nuclei from the postmortem midbrain of six patients with idiopathic PD and five matched controls identified approximately 4,000 microglial nuclei distributed among several subpopulations. Pseudotime analysis suggested branching from a P2RY12-high population toward GPNMB-high and HSP90AA1/IL1B-high states enriched for cytokine signaling, unfolded-protein responses, and cellular stress pathways (Smajić et al., 2022). These states partially share markers reported in other neurodegenerative diseases, but the study did not directly measure glycolysis, mitochondrial respiration, or lipid flux. Their relationship to DAM or MGnD remains uncertain because the study did not directly measure metabolic flux, temporal conversion, or lineage continuity.

Metabolic consequences also depend on the structural form of α-syn and the surrounding inflammatory environment. In primary microglia, fibrillar α-syn assemblies amplified from PD patient material induced stronger inflammatory responses than recombinant fibrils generated *de novo*. When patient-derived fibrils were combined with TNF-α and prostaglandin E_2_ to model chronic inflammatory exposure, microglia acquired a distinct metabolomic and transcriptomic state characterized by disruption of the TCA cycle, changes in glutathione and iron pathways, increased SLC7A11 expression, and enhanced glutamate release. Conditioned medium from these cells increased the death of embryonic mouse midbrain dopaminergic neurons, linking amino acid transport and iron retention to excitotoxicity *in vitro* (Yildirim-Balatan et al., 2024). This state differed from microglia exposed to α-syn fibrils alone and illustrates how inflammatory context can redirect metabolic responses to the same protein aggregate.

Glycolysis-derived metabolites may further stabilize inflammatory programs. In MPTP-treated mice, A53T α-syn transgenic mice, substantia nigra LPS-injection models, and stimulated primary mouse microglia, increased glycolysis and lactate accumulation were accompanied by elevated histone lactylation. H3K9 lactylation was enriched at the Slc7a11 promoter, and inhibition of glycolysis, lactate production, p300/CBP, or SLC7A11 reduced microglial inflammatory responses and dopaminergic neuronal injury (Qin et al., 2025). Because several of these experiments used LPS or MPP^+^ rather than aggregated α-syn, the findings support a glycolysis–lactate–epigenetic mechanism across experimental parkinsonism models rather than an α-syn-specific pathway.

Lipid-droplet remodeling represents another PD-associated response. Single-nucleus analyses identified an ACSL1-high microglial population associated with PD, while experiments in primary mouse microglia and BV-2 cells showed that endoplasmic-reticulum-localized ACSL1 promoted acyl-CoA production and lipid-droplet biogenesis. ACSL1 and TBK1 formed a positive-feedback circuit involving Nrdp1-dependent K63 ubiquitination of TBK1 and NF-κB-dependent ACSL1 transcription. Microglia-targeted ACSL1 knockdown attenuated lipid-droplet accumulation, inflammatory activation, dopaminergic neuronal loss, and motor deficits in LPS- and MPTP-based mouse models (Han et al., 2025); complementary observations were obtained in A53T α-syn mice (Han et al., 2025). These results provide intervention-based evidence for a functional lipid pathway, although toxin and inflammatory models do not fully reproduce idiopathic PD.

Iron handling can connect microglial activation to neuronal ferroptosis. In rotenone-treated mice, microglial CR3 and its ligands were increased alongside abnormal iron accumulation. CR3 deletion reduced NOX2-dependent reactive oxygen species, neuronal iron deposition, lipid peroxidation, dopaminergic neuronal ferroptosis, and neuroinflammation (Wang et al., 2024). In BV-2–SH-SY5Y conditioned-medium experiments, CR3 silencing in microglia similarly reduced neuronal iron accumulation and ferroptotic injury (Wang et al., 2024). This mechanism should be distinguished from direct inflammasome activation: The demonstrated downstream event was microglia-mediated neuronal iron loading and ferroptosis.

Genetic PD models reveal additional metabolic programs not captured by α-syn exposure alone. Human iPSC-derived microglia from patients carrying LRRK2-G2019S exhibited increased glycolysis, altered mitochondrial respiratory capacity, and reduced incorporation of glucose-derived carbon into serine biosynthesis. These cells also showed increased inflammatory signaling and phagocytosis. When incorporated into human midbrain organoids, LRRK2-G2019S microglia promoted dopaminergic neuronal loss, whereas glycolytic inhibition with oxamic acid reduced microglial inflammation and neuronal injury (Kurniawan et al., 2025). This study links patient genotype, isotope-resolved metabolism, and neuronal consequences, but its findings may be specific to LRRK2-associated PD rather than generalizable to all sporadic cases.

Collectively, PD-associated microglial metabolism involves coordinated changes in glycolysis, mitochondrial quality control, lactate-dependent epigenetic regulation, serine synthesis, glutathione and glutamate handling, lipid-droplet turnover, and iron metabolism. Acute glycolytic activation may support migration and aggregate processing in some models, whereas prolonged or genetically altered metabolic states can impair α-syn uptake and increase excitotoxic, inflammatory, or ferroptotic injury. Human single-nucleus studies identify multiple stress- and immune-associated microglial populations, but direct metabolic measurements are still required to link these transcriptional states to the glycolytic, mitochondrial, lipid, amino acid, and iron-related mechanisms defined in experimental models.

### 5.3 Amyotrophic lateral sclerosis

Amyotrophic lateral sclerosis (ALS) is a fatal neurodegenerative disorder characterized by progressive degeneration of upper and lower motor neurons, leading to muscle weakness, paralysis, and respiratory failure (Yuan et al., 2024). Although pathogenic variants in SOD1, C9orf72, TARDBP, and other genes contribute to subsets of ALS, motor-neuron degeneration also depends on non-cell-autonomous interactions among microglia, astrocytes, neurons, and peripheral immune cells (Boillée et al., 2006; Lall et al., 2021). Evidence for microglial metabolic remodeling in ALS is less extensive than that available for AD or PD, but emerging studies implicate redox metabolism, glycolysis, iron handling, glutamate production, lipid-associated signaling, and ferroptotic stress. These pathways generate heterogeneous responses rather than a uniform transition between classical inflammatory phenotypes.

Studies in SOD1 mutant mice first demonstrated that microglial responses change with disease stage. Microglia isolated from presymptomatic SOD1^G93A mice expressed factors associated with neuronal support, whereas microglia collected near end stage exhibited stronger inflammatory and neurotoxic activity in motor-neuron coculture experiments (Liao et al., 2012). However, subsequent RNA sequencing of acutely isolated spinal-cord microglia showed that SOD1^G93A microglia simultaneously expressed potentially protective and detrimental genes and differed from LPS-stimulated, SOD1^WT, and conventional M1/M2 profiles. These changes were detectable across presymptomatic, onset, and late disease stages, indicating an ALS-associated state that cannot be represented adequately by a binary phenotype or a simple protective-to-toxic sequence (Chiu et al., 2013).

Mutant SOD1 can also alter microglial redox signaling. In BV-2 microglial cells expressing SOD1^G93A, SOD1^L8Q, or SOD1^G10V, TLR2 stimulation increased NADPH oxidase-dependent reactive oxygen species production and TNF-α release compared with wild-type SOD1 controls. Conditioned medium from these cells increased neuronal toxicity, linking mutant SOD1, innate immune signaling, and redox stress *in vitro* (Liu et al., 2009). Complementary work showed that mutant SOD1 disrupted the redox-sensitive interaction between SOD1 and Rac1, thereby prolonging Rac1-dependent NADPH oxidase activity in mutant SOD1-expressing cellular systems and transgenic mice. NADPH oxidase inhibition reduced glial toxicity and extended survival in the mouse model, although the *in vivo* intervention was not restricted to microglia (Harraz et al., 2008). These studies support abnormal ROS production as a component of SOD1-associated glial dysfunction but do not establish a generalized glycolytic program across all ALS genotypes.

Iron metabolism provides a more direct connection between microglial metabolic activity and excitotoxic stress. Analysis of spinal cords from 12 patients with sporadic ALS and 12 age-matched controls identified increased soluble iron, ferritin, and glutaminase-C, together with reduced ferroportin. Ferritin, ACO1, TACE, TNF-α, and glutaminase-C immunoreactivity was localized predominantly to microglia. In BV-2 cells, ferric ammonium citrate increased intracellular iron, TNF-α release, and extracellular glutamate. Pharmacological inhibition suggested that iron-induced glutamate release involved ACO1 and a TACE–TNF-α–glutaminase pathway, while hepcidin reduced ferroportin expression (Niida-Kawaguchi et al., 2020). Although the mechanistic experiments used an immortalized murine cell line, the combination of human tissue and intervention-based cell experiments supports a microglial iron–glutamate pathway in sporadic ALS.

Iron accumulation may also produce ferroptotic or sublethal lipid-peroxidation stress in microglia (Fu et al., 2023; Jiao et al., 2022). In a human iPSC-derived neuron–astrocyte–microglia triculture, microglia displayed the strongest transcriptional response to iron and were particularly susceptible to ferroptosis. Iron exposure generated a ferroptosis-associated microglial signature that overlapped with signatures detected in postmortem ALS spinal cord, and removal of microglia delayed iron-induced neuronal injury. A genome-wide CRISPR screen identified SEC24B as a regulator of microglial ferroptosis (Ryan et al., 2023). In a separate study combining microglia–astrocyte–neuron cultures, human sporadic ALS spinal cord, and SOD1^G37R mice, sublethal ferroptotic stress in microglia initiated an inflammatory cascade that converted astrocytes into a neurotoxic state. Treatment of SOD1^G37R mice with the CNS-penetrant ferroptosis-modulating compound Cu^II(atsm) attenuated ferroptosis-associated and neurotoxic glial signatures and was neuroprotective (Liddell et al., 2024). These findings indicate that microglia can mediate the effects of iron and lipid peroxidation without necessarily undergoing complete ferroptotic cell death.

C9orf72-associated ALS provides direct human evidence for genotype-dependent microglial metabolic remodeling (Masrori et al., 2025; Mearelli et al., 2026). Human iPSC-derived motor neurons, astrocytes, and microglia generated from three individuals carrying C9orf72 repeat expansions and their corresponding isogenic controls were examined using metabolic flow cytometry, single-cell RNA sequencing, and multicellular culture systems. C9orf72 mutant microglia showed increased glycolytic activity, oxidative stress, and expression of metabolic enzymes, with stronger abnormalities following inflammatory stimulation. Mutant-specific microglial subpopulations were identified by single-cell analysis. In a motor-neuron–astrocyte–microglia triculture, C9orf72-associated changes in microglia altered metabolic and inflammatory responses in surrounding glia and increased motor-neuron vulnerability (Mearelli et al., 2026; O’Rourke et al., 2016). This study provides stronger cell-specific metabolic evidence than earlier C9orf72 knockout studies, but its conclusions apply primarily to repeat-expansion-associated ALS and should not be generalized automatically to sporadic disease.

Additional genetic signaling pathways may influence microglial metabolic adaptation indirectly. In SOD1^G93A mice, inducible deletion of Ager specifically in microglia altered disease progression in a sex-dependent manner. Transcriptomic analysis linked AGER expression in human ALS spinal cord and the mouse model to pathways involving extracellular-matrix organization, intercellular communication, and lipid metabolism (MacLean et al., 2021). Because metabolic flux was not directly measured, this study supports an association between microglial RAGE signaling and lipid-related transcriptional programs rather than demonstrating a defined metabolic pathway.

Single-nucleus transcriptomics further demonstrates that ALS-associated microglia are spatially and temporally heterogeneous. In SOD1^G93A mice, disease-associated microglia emerged after motor-neuron degeneration, first becoming prominent in the brainstem and spinal cord and increasing during late disease. Selected markers were subsequently confirmed in spinal-cord tissue from patients with sporadic ALS and C9orf72-associated ALS (Chen et al., 2024). These cells expressed genes associated with lysosomal function, lipid processing, phagocytosis, and immune signaling. However, transcriptomic enrichment of metabolic pathways does not demonstrate corresponding changes in glycolytic, mitochondrial, or lipid flux, and pseudotemporal ordering does not establish a lineage sequence.

Collectively, ALS studies identify three prominent components of microglial metabolic dysfunction: mutant SOD1-associated redox and innate immune dysregulation, C9orf72-associated glycolytic and oxidative abnormalities, and iron-linked glutamate release and ferroptotic stress. These mechanisms can alter microglial communication with astrocytes and motor neurons, although their relative contributions are likely to differ among ALS genotypes and affected anatomical regions. Human iPSC-derived systems and postmortem tissue have improved translational relevance, yet direct metabolic-flux measurements in freshly isolated human ALS microglia remain lacking. Therapeutic strategies should therefore account for ALS genotype, disease stage, anatomical region, and the specific metabolic pathway being targeted.

## 6 Discussion

### 6.1 Microglial immunometabolism reflects plasticity rather than fixed polarization

The central conclusion of this review is that microglial immunometabolism should not be viewed as a fixed correspondence between specific metabolic pathways and predefined cellular phenotypes. Instead, metabolic capacity determines the functions that microglia can sustain within a given pathological environment. Glycolysis, mitochondrial respiration, lipid trafficking, amino acid utilization, redox regulation, and lysosomal activity collectively regulate migration, surveillance, phagocytosis, inflammatory signaling, survival, and communication with surrounding neural cells. The functional consequences of metabolic remodeling arise from interactions among pathway activity, organelle function, and pathological demands.

Disease-enriched populations, including DAM, MGnD, LDAM, and TIM, should therefore be compared according to measured metabolic functions rather than arranged into presumed developmental sequences. Although pseudotime analysis and transcriptional similarity may suggest relationships among these states, lineage tracing, longitudinal sampling, and direct metabolic measurements remain necessary to establish temporal transitions. Accordingly, the framework proposed here organizes these relationships without assuming a predetermined developmental order.

This perspective also reconciles apparently conflicting findings across experimental systems. Enhanced glycolysis, mitochondrial activity, or substrate uptake can support the energetic and biosynthetic demands of migration, proliferation, surveillance, and aggregate clearance. Under chronic pathological stress, however, these same pathways may become inefficient, promote harmful metabolite accumulation, or fail to compensate for mitochondrial and lysosomal dysfunction. Likewise, lipid-droplet formation may initially buffer excess fatty acids or cholesterol but later reflect impaired lipid turnover. Glutamine metabolism can also support mitochondrial anaplerosis and phagocytosis or sustain NLRP3-dependent inflammatory signaling, depending on the allocation of glutamine-derived carbon (Terry, 1963; Xu et al., 2025). Metabolic pathways should therefore not be classified as inherently protective or harmful without considering their cellular and pathological context.

### 6.2 Distinguishing adaptive, pathogenic, and reactive metabolic remodeling

A major unresolved question is whether metabolic remodeling initiates microglial dysfunction, represents an adaptive response to pathological stress, or develops secondarily following cellular injury. Current evidence suggests that all three scenarios occur. Acute remodeling may satisfy increased energetic and biosynthetic demands, whereas persistent or dysregulated remodeling can promote inflammatory amplification, oxidative injury, lipid accumulation, excitotoxic metabolite release, or impaired phagocytosis. In other settings, altered metabolic signatures may simply reflect downstream consequences of mitochondrial dysfunction, lysosomal impairment, protein aggregation, or neuronal degeneration.

Distinguishing metabolic association from metabolic causality is therefore essential. Many studies infer metabolic remodeling from glycolytic enzymes, lipid-handling genes, mitochondrial transcripts, or pathway-enrichment analyses. Although these approaches identify candidate metabolic programs, they cannot directly measure substrate utilization, metabolite production, ATP generation, or metabolic flux. Increased glycolytic gene expression does not necessarily indicate enhanced glycolytic ATP production, increased oxygen consumption may reflect inefficient electron transport and reactive oxygen species generation rather than preserved oxidative phosphorylation, and lipid-droplet accumulation alone cannot distinguish altered uptake, synthesis, storage, mobilization, oxidation, or efflux.

Establishing causality requires complementary evidence. First, metabolic pathways should be evaluated using extracellular flux analysis, isotope tracing, metabolomics, lipidomics, or organelle-specific functional assays. Second, genetic or pharmacological manipulation should alter defined microglial functions, including aggregate uptake, migration, cytokine production, glutamate release, redox regulation, or survival. Third, the consequences for neurons, astrocytes, or other neural cells should be demonstrated. Finally, cellular specificity must be confirmed because many metabolic regulators are shared across microglia, infiltrating macrophages, astrocytes, neurons, and peripheral tissues. As only a minority of current studies satisfy all these criteria, transcriptional and spatial associations should be clearly distinguished from intervention-based, cell-specific evidence of causality.

### 6.3 Shared metabolic control nodes produce disease-dependent outcomes

Several immunometabolic regulators recur across Alzheimer’s disease (AD), Parkinson’s disease (PD), and amyotrophic lateral sclerosis (ALS), including glycolytic pathways, lipid metabolism, amino acid metabolism, mitochondrial stress responses, and iron/redox regulation. However, the recurrence of these pathways does not indicate equivalent microglial states or conserved mechanisms across diseases. Instead, they represent context-dependent metabolic control nodes whose functional consequences are shaped by the initiating pathological stimulus, metabolic substrate availability, disease duration, genetic background, organelle integrity, and compensatory capacity.

As summarized in Table 1, shared metabolic alterations should not be interpreted as universal disease mechanisms. The same metabolic program may represent an adaptive response under one pathological condition but contribute to persistent dysfunction under another. For example, metabolic remodeling involving glycolysis, lipid handling, mitochondrial function, or redox regulation may reflect different biological processes depending on the cellular environment and disease stage. Therefore, the presence of a common metabolic signature does not necessarily imply a common upstream driver or therapeutic vulnerability.

**TABLE 1:** Context-dependent roles and evidence strength of major microglial immunometabolic control nodes across Alzheimer’s disease (AD), Parkinson’s disease (PD), and amyotrophic lateral sclerosis (ALS).

Immunometabolic control node	Alzheimer’s disease	Parkinson’s disease	Amyotrophic lateral sclerosis	Cross-disease interpretation
Glycolytic control: mTOR–HIF-1α, PKM2 and lactate signaling	Acute Aβ induces adaptive glycolysis, whereas chronic exposure causes metabolic failure (Baik et al., 2019). Evidence: A, C, F, I.	α-Synuclein activates PKM2-dependent glycolysis and lactate signaling (Lu et al., 2022; Qiao et al., 2019; Qin et al., 2025). Evidence: A, C, F, I.	C9orf72 microglia exhibit enhanced glycolysis without direct evidence for mTOR–HIF-1α regulation (Mearelli et al., 2026). Evidence: H, F.	Glycolysis is a common response, but upstream regulation is disease-specific.
TREM2–APOE signaling and cholesterol handling	TREM2 maintains metabolic fitness and cholesterol homeostasis (Feiten et al., 2026; Haney et al., 2024; Nugent et al., 2020; Ulland et al., 2017). Evidence: H, A, C, F, I.	Evidence for TREM2–APOE-mediated metabolic regulation remains limited.	No direct evidence supports TREM2–APOE-dependent metabolic regulation.	TREM2–APOE is an AD-enriched metabolic pathway rather than a universal regulator.
ACSL1-dependent triglyceride synthesis and lipid-droplet remodeling	APOE4 drives ACSL1-dependent lipid-droplet formation (Haney et al., 2024). Evidence: H, C, F, I.	TBK1–ACSL1 signaling drives lipid-droplet accumulation (Han et al., 2025). Evidence: H, A, C, I.	Direct evidence for ACSL1-dependent lipid metabolism remains limited.	Lipid-droplet accumulation is shared, but upstream drivers differ.
NLRP3, glutamine–glutamate allocation and amino acid metabolism	Glutamine metabolism regulates NLRP3 activation and Aβ phagocytosis (McManus et al., 2025; Zhang et al., 2024). Evidence: A, C, F, I; partial H.	LRRK2-associated serine metabolism and lactate–SLC7A11 signaling predominate (Kurniawan et al., 2025; Qin et al., 2025). Evidence: H, A, C, F, I.	Iron-induced glutamate release predominates in ALS microglia (Niida-Kawaguchi et al., 2020). Evidence: H, C, I.	Amino acid metabolism is shared, whereas regulatory pathways differ.
Mitochondrial metabolic fitness and mitophagy	Chronic Aβ impairs mitochondrial metabolism and mitophagy (Baik et al., 2019). Evidence: H, A, C, F, I.	Persistent α-synuclein disrupts mitochondrial metabolism and mitophagy (Lu et al., 2022). Evidence: A, C, F, I.	C9orf72 and mutant SOD1 impair metabolic fitness, with limited evidence for mitophagy defects (Liu et al., 2009; Mearelli et al., 2026). Evidence: H, A/C, F, I.	Mitochondrial dysfunction is shared, whereas mitophagy defects are best supported in AD and PD.
Iron–NOX2–redox signaling and ferroptotic stress	Microglia-specific ferroptotic mechanisms remain insufficiently established.	CR3–NOX2 signaling promotes neuronal iron deposition and ferroptosis (Ryan et al., 2023; Wang et al., 2024). Evidence: H, A, C, I.	Iron-driven glutamate release and ferroptotic stress promote microglial neurotoxicity (Liddell et al., 2024; Niida-Kawaguchi et al., 2020; Ryan et al., 2023). Evidence: H, A, C, I.	Iron/redox dysregulation is most strongly supported in PD and ALS.

Evidence codes: H, human postmortem tissue or patient-derived/iPSC models; A, animal model; C, primary or established cell culture; F, direct metabolic, isotope-tracing, metabolomic, lipidomic or extracellular-flux measurement; I, genetic or pharmacological intervention. “Limited evidence” indicates that a comparable microglia-specific metabolic mechanism has not been established in the primary studies synthesized in this review; it does not establish the absence of involvement.

A major challenge in interpreting microglial metabolism is distinguishing shared cellular responses from disease-specific regulatory mechanisms. Metabolic pathways are highly interconnected, and their functional consequences depend not only on pathway activation but also on substrate availability, intracellular processing capacity, and interactions with inflammatory and stress-response programs. Genetic factors further modify these metabolic states by altering microglial sensing, lipid processing, mitochondrial homeostasis, and stress adaptation. Thus, identical metabolic signatures may emerge through distinct regulatory routes, whereas similar molecular regulators may produce divergent outcomes across diseases.

Collectively, comparative evidence indicates that shared immunometabolic regulators represent points of convergence rather than universal mechanisms of neurodegeneration. Understanding how pathological context determines the functional output of these control nodes will be essential for developing precise interventions targeting microglial dysfunction.

### 6.4 Therapeutic implications and the challenge of microglial specificity

The context-dependent nature of microglial metabolism has important therapeutic implications. Broad inhibition of glycolysis, mitochondrial metabolism, lipid synthesis, or amino acid utilization is unlikely to achieve sufficient cellular specificity because these pathways are also essential for neurons, astrocytes, oligodendrocytes, infiltrating immune cells, peripheral tissues, and physiological microglial functions. Consequently, systemic inhibition of central metabolic pathways may impair normal cellular homeostasis while suppressing disease-associated responses.

Therapeutic strategies should therefore target specific metabolic defects rather than metabolic activation itself. Depending on the pathological context, effective approaches may include restoring mitochondrial or lysosomal function, improving intracellular lipid trafficking and turnover, redirecting amino acid metabolism, limiting toxic metabolite production or release, and preserving metabolic flexibility during chronic stress. Such interventions should be supported by evidence that the targeted metabolic abnormality contributes directly to disease rather than merely accompanying microglial activation.

The timing of intervention is likely to be as important as the molecular target. Pathways that initially support migration, surveillance, or aggregate clearance may become detrimental after prolonged stimulation, organelle dysfunction, or loss of metabolic capacity, whereas premature inhibition of inflammatory pathways may suppress adaptive responses. The coexistence of multiple microglial states within the same tissue further complicates therapy, as a pathway may be pathogenic in one population but essential for survival or clearance in another.

Achieving microglial specificity will therefore require integrating cellular identity, functional state, anatomical location, and disease stage. Preclinical studies should determine whether interventions act directly on resident microglia or indirectly through neurons, astrocytes, infiltrating macrophages, or systemic metabolism. State-resolved biomarkers will also be required to identify the disease stages and patient populations in which specific metabolic abnormalities are active and therapeutically relevant. Ultimately, successful translation is more likely to depend on state-selective, temporally controlled correction of defined metabolic defects than on sustained inhibition of broadly expressed metabolic pathways.

### 6.5 Human relevance and future research priorities

Human relevance remains a major limitation of the current evidence base. Postmortem single-cell, single-nucleus, and spatial studies demonstrate that human microglia are heterogeneous and include disease-enriched populations associated with lipid handling, antigen presentation, lysosomal function, cellular stress, and inflammatory signaling. However, these datasets primarily provide transcriptional and spatial associations rather than direct measurements of metabolic activity, and are influenced by advanced disease stage, postmortem interval, agonal conditions, tissue dissociation, treatment exposure, and regional sampling.

Human induced pluripotent stem cell-derived microglia enable genetic manipulation, isotope tracing, metabolic-flux analysis, and multicellular coculture. However, they do not fully recapitulate aging, regional specialization, vascular interactions, systemic immune exposure, or decades of progressive pathology. These models should therefore be regarded as experimentally accessible systems for studying selected human genotypes and cellular interactions rather than complete representations of adult brain-resident microglia.

Future studies should integrate direct metabolic measurements with state-resolved molecular profiling. Single-cell and spatial transcriptomics should be combined with isotope tracing, metabolomics, lipidomics, extracellular flux analysis, mitochondrial and lysosomal functional assays, and targeted perturbation within the same experimental systems to determine whether transcriptionally defined states exhibit the predicted metabolic functions.

Longitudinal studies are also needed to distinguish early adaptive remodeling from late metabolic exhaustion or secondary responses to cellular injury. Experimental designs should account for sex, age, anatomical region, genetic background, pathological stage, treatment exposure, and the structural forms of disease-associated protein aggregates. Although immortalized microglial cell lines, neonatal rodent microglia, acute LPS stimulation, and peripheral macrophages remain valuable for mechanistic studies, their findings require validation in physiologically relevant adult human systems before being generalized to neurodegenerative diseases.

In conclusion, microglial immunometabolism links pathological exposure to the cellular capacity for surveillance, aggregate clearance, inflammatory signaling, redox regulation, and intercellular communication. The effects of metabolic pathways cannot be inferred solely from increased or decreased activity. Future progress will depend on direct, cell-specific, state-resolved, and temporally precise measurements of metabolic function rather than reliance on fixed phenotypic classifications or transcriptomic inference. Ultimately, the key therapeutic challenge is not whether microglial metabolism should be activated or suppressed, but which metabolic function should be restored, redirected, or limited in a defined microglial population at a specific stage of disease.
